# “I Get That Spirit in Me”—Mentally Empowering Workplace Health Promotion for Female Workers in Low-Paid Jobs during Menopause and Midlife

**DOI:** 10.3390/ijerph17186462

**Published:** 2020-09-04

**Authors:** Marjolein Verburgh, Petra Verdonk, Yolande Appelman, Monique Brood-van Zanten, Karen Nieuwenhuijsen

**Affiliations:** 1 Department of Public and Occupational Health, Amsterdam Public Health Research Institute, Amsterdam UMC University of Amsterdam, Meibergdreef 9, P.O. Box 22660, 1100 DE Amsterdam, The Netherlands; k.nieuwenhuijsen@amsterdamumc.nl; 2 Department Ethics, Law and Humanities, Amsterdam Public Health Research Institute, Amsterdam UMC VU University, Boelelaan 1089a, 1081 HV Amsterdam, The Netherlands; p.verdonk@amsterdamumc.nl; 3 Department of Cardiology, Amsterdam UMC VU University, Boelelaan 1117, P.O. Box 7057, 1007 MB Amsterdam, The Netherlands; y.appelman@amsterdamumc.nl; 4 Department of Gynecology, Amsterdam UMC University of Amsterdam, Meibergdreef 9, P.O. Box 22660, 1100 DE Amsterdam, The Netherlands; m.brood@amsterdamumc.nl; 5 Department of Gynecology, The Netherlands Cancer Institute, Plesmanlaan 121, P.O. Box 90203, 1006 BE Amsterdam, The Netherlands

**Keywords:** women, midlife, menopause, work, low-paid jobs, workplace health promotion, intervention

## Abstract

During menopause and midlife, female workers, particularly those in low-paid jobs, experience more occupational health problems than other groups of workers. Workplace interventions are often lacking, however. In the Netherlands, a workplace health promotion intervention—the work–life program (WLP)—has been developed to support female workers. Here, we tailored the WLP to the needs of female workers in low-paid jobs working at Amsterdam University Medical Center. In an exploratory mixed-methods study with a convergent design, among 56 participants, we used questionnaires before and after the intervention and semi-structured, in-depth interviews to address the following research question: What is the impact of the WLP on the women’s health and work functioning? Our quantitative data showed that menopausal symptoms improved significantly after the WLP. Our qualitative data, derived from 12 participants, showed that the WLP initiated a process of mental empowerment that initiated positive changes in four domains: behavior, physical health, mental wellbeing, and in the workplace. Taken with caution, our findings suggest that the WLP mentally empowers female workers to make choices that enhance their health and wellbeing, both at work and in their private lives, as summarized in the quote of one participant: “I get that spirit in me!”.

## 1. Introduction

As in other Western countries, in the Netherlands almost half of all paid work is done by women [1,2,3]. Of these working women, more than a third are in midlife, i.e., aged 45 or older [3,4]. Female workers are known to be concentrated in specific sectors of the labor market, and in specific occupations within these specific sectors, mainly in health care and education [5]. Female workers are often confronted with unfavorable working conditions, such as a low degree of autonomy and high emotional demands, as well as workplace violence [6,7]. Moreover, women’s lower work ability compared to men’s has been explained by a gender imbalance in domestic work [8]. Women in general are known to be absent due to sickness more frequently than men [7,9,10]. Apart from the negative consequences for women themselves when they call in sick, this has economic consequences for the employer and the specific sectors in which women work.

Within these traditionally female sectors, women are more likely to work in low-paid jobs than men [5]. For instance, in the health care sector, they are likely to be cleaners, patient food service assistants, and ancillary workers [5]. Women in low-paid jobs are also more likely to be faced with unfavorable physical and social working conditions which put them at greater risk of poor health, and poor work functioning. Examples of unfavorable physical working conditions include physically demanding work and inflexible working hours [11]. Examples of unfavorable psychosocial working conditions include high levels of work stress and a low degree of autonomy [11]. Furthermore, unhealthy lifestyle behaviors, such as smoking, poor diet, and physical inactivity are more common in people with a lower socioeconomic position (SEP) [12].

On top of the unfavorable working conditions among female workers in low-paid jobs comes these women’s transition to midlife, which includes both a biomedical transition and a life phase transition. By biomedical transition we mean the menopause that refers to the twelve-month absence of a menstrual cycle [13]. What women experience is not just the absence of a menstrual cycle, but they experience a transition lasting for many years that is associated with a wide range of symptoms [13]. These menopausal symptoms include hot flashes, sleep disturbances, night sweats, anxiety, weight gain, loss of sexual desire, anxiety, depression, mood swings, irritability, memory loss, poor concentration, and joint pain [1,14]. These menopausal symptoms can be aggravated by an unhealthy lifestyle [15], which is more common among women with a lower SEP. Factors related to a lower SEP are associated with a longer duration and severity of menopausal symptoms [16,17,18,19,20]. Limited research on this topic has shown that menopause is negatively associated with work outcomes [14,21,22,23,24,25]. Previously, it has been reported that female workers with unfavorable physical and psychosocial working conditions experience in all likelihood additional difficulties related to menopausal symptoms at work [14]. Menopause is also associated with an unfavorable risk factor pattern regarding blood pressure, diabetes mellitus, weight gain, fat distribution, and cholesterol levels [26]. In addition to the menopause, the transition to midlife is considered to be a life phase transition. During this transition, women have to deal with changing social factors which may include informal care responsibilities for chronically ill or disabled parents or partners, the death of parents or a partner, raising adolescent children, children leaving home, potential divorce, and potentially financial insecurity after going through divorce [1,27]. A recent study found that the interference of social factors of women’s private lives, typical for midlife, represents a central risk factor for work ability [8]

The workplace set-up does not allow women sufficient space to cope with the transition to midlife. Despite the fact that female workers in low-paid jobs are likely to experience a greater impact on their health and work functioning, workplace health promotion interventions to support female workers during menopause and midlife, particularly those in low-paid jobs, are not yet available. Part of the reason is that, in general, employers have little knowledge of gender-related health issues typical of midlife, and do not know how to contribute to a more healthy work environment for female workers during menopause and midlife [1,14].

Studies that have evaluated interventions focused on midlife and work are scarce [28]. We only found two studies in which a work-related health intervention for menopausal female workers from various job groups was examined. The first is a Japanese study by Ariyoshi (2009) which mainly evaluated the role of an occupational health nurse as part of new health system aimed at supporting menopausal female workers in a media company (e.g., journalists, administrative and sales workers) [29]. A number of female workers experienced a decrease in menopausal symptoms after implementation of the interventions [29]. The second study is a British randomized controlled trial by Hardy et al. (2018) which aimed to investigate the impact of a self-help cognitive behavior therapy intervention in female workers from eight organizations in both the private and public sectors who were having problematic hot flashes and night sweats [30]. This study showed that the intervention had a positive effect on the frequency and problem rating of hot flashes and night sweats, and on several other outcome measures [30].

Two other studies on work-related health interventions examined support to menopausal female workers delivered outside the context of the workplace. The first study is a Dutch retrospective cohort pilot study by Geukes et al. (2019), in which the authors examined the relationship between the improvement in severe menopausal symptoms and work ability. Participants were female workers who attended a menopause clinic for the first time. The intervention included consultations with a specialist nurse who provided advice on menopausal symptoms and lifestyle changes. The results of Geukes et al.’s study showed that treatment aimed at improving menopausal symptoms in severely symptomatic female workers was associated with improvement in menopausal symptoms and an improvement in ability to work [31]. The second study is a Finnish randomized controlled trial study by Rutanen et al. (2014), in which the authors examined how physical exercise—mainly aerobics—related to daily mental and physical strains and work ability in female workers with menopausal symptoms [32]. When compared with the control group, after the intervention this study showed an improvement in mental resources and a decrease in physical strain [32]. However, the results show no statistically significant difference in work ability between the intervention and control group.

However, the interventions evaluated in the few studies mentioned above do not address the broader problems faced by women in midlife. In contrast, these studies focus mainly on menopausal symptoms in relation to work in female workers in various job groups. If issues in midlife are not just about medical aspects such as menopause, what should a work-related intervention aimed at supporting female workers during midlife look like? In this study, we offered an intervention with an integral approach that aimed at supporting female workers in the broader difficulties they may encounter in midlife, and one that takes into account the needs of the specific target group. In the Netherlands, a private company called HealthyWoman has developed a workplace health promotion intervention—the work–life program (WLP)—aimed at supporting female workers during menopause and midlife in making choices that will enhance their health and wellbeing in both their working and private lives. The WLP applies an integral approach which encompasses an intake session to explore the needs of the participant, health education on menopause and lifestyle, coaching to improve work–life balance, and physical training. The WLP had previously been implemented in multiple Dutch organizations employing many female workers in low-paid jobs, however, scientific evaluation of the implementation, including the impact of the intervention, had not yet taken place.

If we evaluate implementation, and the impact of the WLP on women’s health and work functioning, we could gain insight into how to support female workers in low-paid jobs during menopause and midlife. By gaining more insight into the implementation of these women’s support, workplace health promotion can be designed and implemented in the workplace in a targeted manner, which may lead to less sick absenteeism among this target group. Therefore, we implemented a version of the WLP tailor-made to the needs of female workers aged from 45 to 60 in low-paid jobs, such as food assistants and cleaners, who worked at two locations at the Amsterdam University Medical Center. In an explorative study, we addressed the following research question: What is the impact of the WLP on women’s health and work functioning? This exploratory study is part of a larger implementation study in which, in addition to examining the impact of the WLP, we also conducted a process evaluation of the implementation. The purpose of this process evaluation is to define important aspects of how to reach and engage a culturally diverse group of midlife women in low-paid jobs. The results of the process evaluation will be reported in our separate article (Verburgh et al., Forthcoming).

Using a mixed-methods study design, we gathered quantitative and qualitative data. Self-reported questionnaires were used to determine changes in five outcome variables after the intervention. The variables were a need for recovery after work, work functioning, menopausal symptoms, quality of life, and work ability. Semi-structured interviews conducted after the intervention were used to assess changes in health and work functioning perceived by the female workers.

The quantitative analysis showed a significant improvement in menopausal symptoms, but no significant change in the four other outcome variables. The qualitative analysis of perceived changes in women’s health and work functioning revealed that the WLP initiated a process of mental empowerment that had a positive impact on the participants’ behavior, physical health, mental wellbeing, and the workplace.

## 2. Materials and Methods

### 2.1. Design

In a mixed-methods study design, we employed a convergent design [33], in which we conducted two parallel studies, i.e., we collected both quantitative and qualitative data. We merged the results from the quantitative and qualitative data in order to enrich our understanding beyond what would be possible from quantitative or qualitative results alone. For the quantitative section of our study, we applied a pre-test–post-test study, i.e., self-reported questionnaires, in order to determine changes in the five outcome measures related to health and work functioning. Since this mixed-methods study is part of a larger implementation study in which we implemented the WLP, as a tailor-made intervention, in a “real-world” setting, we did not include a control group. A randomized control trial (RCT) requires a standardized intervention being implemented uniformly within a homogenous target population [34], but this was not the case in our study. Therefore, we also conducted a qualitative study, i.e., semi-structured, in-depth interviews, in order to understand the perceived changes in health and work functioning. In this way, the qualitative data enriched the quantitative data and deepened our understanding of how participation in the intervention has led to changes in women’s health and work functioning among female workers in low-paid jobs.

### 2.2. Participants and Recruitment

We aimed to include 75 participants in the intervention and study activities. A power analysis based on data that stemmed from a preventive intervention among informal care providers, revealed that at least *n* = 51 participants with full data are needed to detect a statistically significant improvement on the primary outcome measure need for recovery (NFR) with a minimum power of 80% and a two-sided alpha of 0.05. We recruited female workers aged between 45 and 60 years working in low-paid jobs (e.g., patient food service assistants and cleaners)—an annual income of minimum EUR 22.250 and maximum EUR 35.245 at a full time basis— at Amsterdam University Medical Center. The majority of this group of female workers do not work on a full-time basis. As we had the help of an interpreter, female workers who were not able to speak Dutch were able to be included in the quantitative study. We recruited participants in collaboration with the Human Resources (HR) department of the organization. Participation in the intervention and study activities was voluntary. We recruited through various channels, but a personal invitation letter sent by the HR department was the first step in the recruitment process. For female workers who had difficulty with the Dutch language, in collaboration with line managers, we simultaneously organized small meetings to exchange information about the intervention and study. This group of workers did not receive the personal invitation letter. Next, we organized several information meetings for all female workers from the target population in which an inspirational speaker explained the relationship between menopause and work, and the developer of the intervention introduced the intervention itself. A representative of the HR department emphasized the importance of this study in the opinion of the organization, and explained the privacy terms and the voluntary nature of participation. The first author then introduced the study and explained its aims and the methods of measurement. Furthermore, we involved female workers from the target group who were willing to share their experiences with the menopause. In addition, we hung up posters in the hospital and actively distributed flyers around various wards.

Since we aimed to collect experiences of perceived changes through participation in the intervention, participants in the qualitative study comprised those women who completed the entire intervention (including pre-test–post-test study). In total, we planned to include twelve participants in the semi-structured in-depth interviews. We selected participants using a purposive sampling strategy which is a non-random way of ensuring that particular categories are included in the study sample [35]. We selected participants with different characteristics at baseline: age, ethnicity, educational level, type of contract, living situation and menopausal status. Participants who were not able to speak Dutch were excluded from the qualitative study, but participants who could speak a little Dutch were included. In the interviews with this group of participants, we spoke slowly and repeated questions when necessary.

### 2.3. Intervention

**Figure 1:**
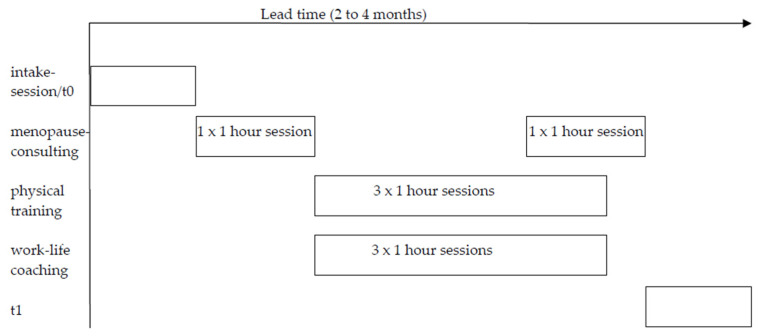
Work–life program.

The intervention was a pre-existing workplace health promotion intervention—the work–life program (WLP) which is aimed at supporting menopausal female workers during midlife in making choices that enhance their health and wellbeing. Before starting the WLP, an intake session was held in which the WLP was explained in detail, background information on the participant was collected, and the WLP was customized to the needs of the participant. The WLP consisted of three components spread over eight sessions of one hour with a planned lead time of a few months (Figure 1). There was no fixed schedule, because the planning depended on practicalities, such as the availability of an intervention provider and participant. However, the order of sessions did not change: a menopause consultation, followed by work–life coaching sessions and physical training sessions simultaneously, and a second menopause consultation. Two one-hour menopause consultations focused on education about menopause symptoms, how these may affect working and private life, and what can be done to ameliorate or cope with these symptoms. Medical interventions such as menopausal hormone therapy were discussed at the discretion of the participants. Participants who were contemplating menopausal hormone therapy were referred to their general practitioner, who is able to refer to gynecologists or other specialist care. The importance of healthy dietary habits and physical activity was also explained during these meetings. Finally, a health check (weight, height, blood pressure and waist circumference) was conducted and the results were discussed. Counseling of women, including individualized educational strategies, was found to be effective in improving healthy behaviors of perimenopausal women [36]. Counseling has further been advocated as an effective strategy to reduce vasomotor symptoms, as an alternative for or addition to hormone replacement therapy [37]. Counseling further aligns well with proposed employer-level actions thought to be helpful by working women [14]. They expressed the need to receive information and advice about coping strategies at the workplace. Three one-hour work–life coaching sessions focused on individuals to improve their work–life balance. A coach supported the participant in acquiring insight into their personal needs and goals, and into which factors contribute to a work–life balance. Together, the coach and the participant decided on strategies to improve the work–life balance of the participant. Work–life coaching sessions are part of the WLP, because stress from either work or private sources is related to a lower quality of life during and after menopause [38]. The three one-hour physical training sessions were given, comprising walk training, or personal training if the participant preferred this type of physical activity. The physical training sessions were the only component that could be given individually or group-based. These training sessions were aimed at helping the participant to understand the health benefits of physical activity, and to encourage them to find meaningful ways of being active in their daily lives. Offering physical training sessions as part of the WLP is based on the findings that physical inactivity is associated with lower physical and mental health during and after menopause [36,37,38] Hence, the WLP was a tailor-made intervention adapted to the specific needs of the participants. 

### 2.4. Data Collection

The research proposal was approved by the Medical Research Ethics Committee of the Amsterdam University Medical Center, location VUmc, in the Netherlands (2018.635, 28 November 2018). A comprehensive evaluation was not required since this study was not subject to the Medical Research Involving Human Subjects Act. All participants gave their written informed consent during the intake session prior to taking part in the pre-test–post-test study, intervention, and semi-structured in-depth interviews. The inclusion of participants started in December 2018, data collection started in February 2019, and finished in January 2020. Individual data were treated confidentially by the researchers (MV and KN), and were not shared with the employer, with the exception of a select group of participants. Information about their participation was shared at their own request.

#### 2.4.1. Quantitative Study

##### Procedure

We used quantitative methods, i.e., self-reported validated questionnaires (t0 and t1), to determine whether there were changes in the study population in five outcome variables after the intervention. The variables were: the need for recovery after work, work functioning, menopausal symptoms, quality of life and work ability. Participants filled out the questionnaire at t0 during the intake session in the presence of the first author, who assisted if questions were not clear to the participant. An interpreter was available for those participants unable to read Dutch throughout the entire intervention (except for physical training). The questionnaire at t1 was sent to the participant’s home address within the first two weeks after the intervention was finished. If a participant was unable to read Dutch then an appointment was made with the first author and an interpreter. If a participant had difficulty reading Dutch, only the first author assisted with filling out the questionnaire at t1. We offered a EUR 50 gift voucher as an incentive to participants who completed both questionnaires.

##### Measures

Participant characteristics: In the questionnaire at t0, we obtained information on participant characteristics including the following variables: age, ethnicity, educational level, subjective social status (SSS), living situation, main wage earner, type of work contract, and informal care responsibilities. In the questionnaire at t1, we obtained information on menopausal status. We obtained information on age in years.

We obtained information about ethnicity by asking where the participant was born, and where both parents were born. Based on the Dutch Central Bureau of Statistics (CBS) criteria [39], we defined ethnicity according to the country of the birth of the participant as well as her parents [40]. A participant was considered as belonging to an ethnic minority group if she fulfilled one of the following criteria: being born abroad and having at least one parent born abroad, or being born in the Netherlands but both parents being born abroad. A participant was considered ethnic Dutch if both parents were born in the Netherlands.

We assessed educational level by the participant’s highest level of education (either in the Netherlands or abroad). Based on the Dutch Central Bureau of Statistics (CBS) criteria [41], we defined low educational level by completion of primary school, lower vocational education, and lower secondary school; intermediate educational level by completion of intermediate vocational education and upper secondary school; higher educational level by completion of upper vocational education and university.

We measured subjective social status (SSS) with the MacArthur scale [42], and it can be defined as an individual’s perception of his or her own position in the socioeconomic hierarchy [42]. This instrument consists of a single question (“Think of this ladder as representing where people stand in our society (the Netherlands). At the top of the ladder are the people who are the best off, those who have the most money, most education, and best jobs. At the bottom are the people who are the worst off, those who have the least money, least education, and worst jobs or no job [43].” A 10-point response scale was illustrated in a ladder on which the participant could draw a cross (0 = worst off, 10 = best off).

We assessed living situation by asking what participant’s living situation is (1 = alone, 2 = with partner, 3 = with partner and children, 4 = alone with children, 5 = other).

We obtained information about the main wage earner by asking if the participant is the main wage earner in the household (1 = yes, 2 = no, my partner and I earn about the same, 3 = no, my partner is main wage earner).

We assessed type of employment contract by asking what type of employment contract participants have (1 = full-time, 2 = part-time).

We obtained information about informal care responsibilities by asking if the participant provide informal care on a weekly basis (1 = yes, 2 = no).

We assessed menopausal status by asking whether the participant still menstruates (1 = yes, I still menstruate as often as before (no cycle changes) (pre-menopause; 2 = yes, but I menstruate more often/my cycle is irregular (≥7 days difference between consecutive periods) (early-peri menopause; 3 = yes, but I menstruate less often and skip periods (≥60 days between 2 periods) (late-peri menopause); 4 = no, I have not menstruated for more than 12 months (post-menopause); 5 = I do not know, because I use hormonal contraception (unknown)). The classification for menopause status is based on the STRAW+10 staging system [44].

Outcome variables: We used the following questionnaires to measure the five outcome variables at pre-test (t0) and post-test (t1). Need for recovery after work (NFR) was the primary outcome variable, and we measured this variable with the Need for Recovery Scale questionnaire [45]. The NFR scale consists of 11 items, which uses a dichotomous response scale. The total score ranges from 0 to 100 points (lower representing better functioning).

We measured work functioning with the weighted composite work-functioning questionnaire [46] This questionnaire comprised 49 items divided into four domains: work performance (18 items), NFR (11 items) (primary outcome variable), quantity of work (5 items), and capacity to work (15 items), which uses various response scales [46]. We converted the domain scores to a 0–100 scale, and used these scores to calculate the total work functioning scale ranging from 0 to 100 points (lower representing better functioning) [47].

We measured menopausal symptoms with the Dutch version of the Greene Climacteric Scale (GCS) which is a widely used self-report measure of menopausal symptoms [23,48]. The GCS consists of 21 items, divided into various domains: psychological (eleven symptoms), subdivided into anxiety (7 symptoms) and depression (5 symptoms), somatic (7 symptoms), vasomotor (2 symptoms), sexual (1 symptom), which uses a 5-point response scale (0 = not existing, 1 = sometimes, 2 = often, 3 = very often) [23]. The total GCS ranged from 0 to 63 points (lower is better) [23].

We measured quality of life with the Dutch version of with the Medical Outcomes Study Short Form 12 (SF-12) [31]. This questionnaire consists of 12 items which measure physical and mental health, which uses various response scales. The total scale ranged from 0 to 100 points (higher is better).

We measured Work ability with the work ability score (WAS) which is the first question of the Work ability Index (WAI). The WAS measures the general work ability compared to lifetime best [49]. The WAS ranged from 0 to 10 (higher is better), 0–5 points is poor, 6–7 points is moderate, 7–8 is good and 9 is excellent.

#### 2.4.2. Qualitative Study

##### Procedure

We used the qualitative method of semi-structured, in-depth interviews to determine the perceived changes in health and work functioning after the intervention. These interviews were conducted after the intervention by the first author (MV) who is a trained medical anthropologist, and who had already built up a relationship with the participants at the beginning of the intervention, which was conducive to trust. The interviews had a broader scope than the items used for this article, but the topic list relevant to this article was based on the five outcome variables from the questionnaires in the quantitative study. We broadened the focus of the outcome variables to include larger themes, such as lifestyle, health and work. In Table 1, the topic list relevant to this article is outlined. The first author conducted each interview in Dutch. Each interview was held either in a private room in the participant’s workplace or in the hospital restaurant (depending on the participant’s preference). The average length of an interview was an hour. We offered a EUR 25 gift voucher as an incentive to participate in the interview.

### 2.5. Data Analysis

#### 2.5.1. Quantitative

We reported descriptive information on participant characteristics as means, standard deviation, or frequencies (%).

Differences between pre-test (t0) and post-test (t1) were analyzed by either paired-sample *t*-tests or Wilcoxon signed-rank tests. For each outcome variable, we examined the distribution of the difference between pre-test (t0) and post-test (t1) using the Shapiro–Wilk Test. For continuous outcomes that were normally distributed, we used paired-sample *t*-tests to determine whether statistically significant difference occurred between outcomes of pre- and post-test. For outcomes that were not normally distributed, we used the Wilcoxon signed-rank test. We set the significant level at 0.05. We used SPSS statistics 25.0.

Furthermore, we analyzed effect sizes to determine the relevance of outcome variables that changed significantly. We calculated the Cohen’s d effect size [50] by determining the mean difference between pre- and post-test, divided by the pooled standard deviation [51]. For Cohen’s d, a score of 0.2–0.5 can be considered a small effect, 0.5–0.8 a medium effect, and >0.8 a large effect [50]. We used Review Manager 5.4.

#### 2.5.2. Qualitative

We recorded and transcribed all interviews verbatim. During the first phase, two researchers (MV and KN) individually coded the first three interviews deductively and inductively, then compared and discussed these codes to reach an agreement; this resulted in a coding scheme. Subsequently, only the first author deductively and inductively coded subsequent interviews in accordance with the scheme, adapting it when a new code came up [52]. Another author (KN) checked the new codes, after which we retained the codes for which agreement had been found, which led to a new version of the coding scheme. In the second phase, we sorted the codes into themes. To support our findings in the results section, we included quotes from the interviews with participants. The quotes in the results section of this article were translated from Dutch by a native English speaker. We used MAXQDA 2020, VERBI Software, Berlin, Germany.

## 3. Results

### 3.1. Quantitative

#### 3.1.1. Participant Characteristics

The total number of participants in the pre-test–post-test study was 70 at pre-test (t0), and 56 at post-test (t1). Fourteen participants stopped participating in the WLP. Participant characteristics are listed in Table 2. More than half of the participants had a migration history that varied greatly—21 different migratory backgrounds. Almost all participants had a low or intermediate educational level. Participants scaled themselves relatively low on the socioeconomic ladder at a mean of 4.7 (1.5) on a scale of 0–10. More than one-third of the participants did not live with a partner, and over half the participants were the main wage earner in the household. Over half of the participants had a part-time employment contract. About one-fifth of the participants had informal care responsibilities on a weekly basis. Two-thirds of the participants were post-menopausal women.

#### 3.1.2. Pre-Test–Post-Test Outcomes

Table 3 shows that NFR did not significantly decrease between pre- and post-test. This also applies to all other subscales of work functioning, including the total sum score.

The total score on menopausal symptoms significantly decreased after participation in the WLP (from 17.9 to 14.5, *p* = 0.000). All subscale scores significantly decreased after participation in the WLP, with the exception of anxiety (from 4.8 to 4.4, *p* = 0.099), and sexual dysfunction (from 0.9 to 0.8, *p* = 0.438). The effect size of the total sum score on menopausal symptoms including all subscale scores with a significant effect was between 0.2 and 0.5, which means a small effect.

Regarding quality of life, the score on both outcome variables did not change between pre- and post-test.

Work ability improved (from 7.0 to 7.4), but was not statistically significant (*p* = 0.072).

### 3.2. Qualitative

The WLP initiated a process of mental empowerment in most participants, whereby they have learned to reflect more on their own needs. This helped them to make more conscious choices that benefit both their health and work functioning. We have identified this process of mental empowerment as the central theme of the analysis, and it is reflected in the quote: “*I get that spirit in me*!” from a 49-year-old cleaner with a Ghanaian migratory background. In her culture, it is not customary to talk about personal issues. For instance, she had lost interest in sex with her partner, but did not talk about this issue with him. After the WLP, she felt mentally empowered to start discussing this issue with her partner in order to relieve the tension between them. In this account of the qualitative findings, we define mental empowerment as a form a self-efficacy, i.e., the belief in one’s ability to exert control over one’s own motivation, behavior, and social environment [53]. When we spoke about these kinds of changes in the interviews, which we relate to mental empowerment in our analysis, almost all participants said that they felt stronger or more free. To illustrate this, we have used quotes from several participants who work as patient food service assistants:

No, I have become stronger. […] So, about me? I am now really super strong. (20190924)

Yes, I have had some really good talks with you. And that has given me a real lift, it has given me the power, the power to keep on going! (20200123)

Liberation haha, yes, a sort of weight has been lifted off my shoulders. (20191127)

Although not for everyone, this process of mental empowerment seems to be associated with positive changes in four domains: behavior, physical health, mental wellbeing, and in the workplace. The importance of mental empowerment is even more clear when listening to the account of a 56-year old laundry operative who had been suffering from menopausal symptoms—mainly difficulty in sleeping, hot flashes and joint pain. In the following quote, she tells that she had learned from the WLP, but had not experienced any changes in her menopausal symptoms:

Yes, you learn, but change...? Nothing has really changed... No, there hasn’t been much change for me. (20191105)

At the same time, she said that she had not changed her behavior regarding exercising and self-care, apart from taking dietary supplements. This example indicates that she did not feel mentally empowered to change certain health behaviors, although this would appear to be a prerequisite for experiencing changes in health and wellbeing. We identified four domains as four sub-themes in the analyses, which we will report below. Participant characteristics are listed in Table 4.

#### 3.2.1. Behavioral Changes

We subdivided ‘behavioral changes’ into health, lifestyle, and social behavior. Changes in health behavior varied from planning rest periods to avoid becoming completely exhausted by the end of the day, to making small adjustments in sleep hygiene. Almost all participants followed the advice of the menopause consultant to use dietary supplements, in particular vitamin D3 and magnesium citrate. These supplements were advised in combination with a calcium-rich diet in order to prevent osteoporosis, and to promote better sleep and reduce muscle cramps. Following the advice of the physical trainer, the majority of the participants said that they had become consciously engaged in changing their posture to improve physical health, such as back pain. This 56-year-old employee in medical administration is now more aware of her posture when walking:

No, but it has had a big effect on me. As I have said before, I often got backache when I’d been walking. Walking is good, so what am I doing wrong? I thought it was just my back. I mean, when you get older your back starts to hurt, but it is probably due to the fact that I was holding myself too straight and stiff, and walking too fast. I don’t know, but now I am much more conscious of how I should walk. (20191111)

Almost all participants stated mentioned that they tried to put new and better posture into practice.

Changes in lifestyle behavior mainly concerned changes in nutrition, such as a more varied diet. However, not everyone discussed nutrition extensively in the WLP, because they already had a healthy diet. A number of participants started exercising more often (or more consistently) due to participation in WLP, for example with the help of a pedometer. This was not successful for everyone, and explanations given included not being able to fit exercising into their busy daily schedule. A 56-year-old laundry operative, for whom integrating exercise into her daily schedule was difficult, initially described herself as lazy. In the following quote, she pictures her daily schedule:

Yes, after six hours [of working] I get home at 3:00 p.m. Normally it is 3:00 p.m., go by the supermarket, and get home at 3:30 p.m. Oh, then I go to the attic, iron, fold, tidy up, clean, go to the kitchen, I’m done at 8:00 p.m. […] And then I don’t feel like going outside anymore. (20191105)

Together, we came to the conclusion that she is not lazy, but has other priorities due to her gendered caring responsibilities at home.

Changes in social behavior involved both work and private life. An example of a change in social behavior is that various participants told us that they had started to share their experiences of the WLP with other women, including colleagues and family members. Topics included advice on dietary supplements and posture; this was particularly true of participants with a non-Western migratory background. In the following quote, a 54-year-old patient food services assistant with a Hindu-Surinamese migratory background explains how uncommon it is in her community to talk about private issues, but started to share her newly acquired knowledge in the WLP with her daughters-in-law:

Then in my women’s group, in our group of Hindustani women, well they don’t talk about themselves. But, with this thingy [WLP] you can talk freely. And also I can be aware now, for my daughters-in-law for example. So I can explain to them, if you are in your 40s, or around 45 or in your 50s, then you can get these symptoms. […] But no-one said: maybe menopause, that … They don’t talk much. I am happy that you have come up with this program and that such things exist in the Netherlands. That we can get on with it. (20200123)

This quote suggests that participants feel the need to share their newly acquired knowledge from the WLP with their social environment, which then has an effect beyond the participants themselves and extends into their communities. In terms of changes in social behavior—specifically in working life—a number of participants said that they now interact differently with their colleagues. To illustrate this, we use a quote from a 54-year-old patient food services assistant who works flexible shifts. She often got furious if she started her shift and the handover from her predecessor was a mess. In the following quote, she reflects on the change she has experienced in her way of interacting:

It all starts with the handover. […] I was someone that was like ‘don’t mess with me’. You know, but now I think what the...? [colleague’s name] is just a colleague, I come to work, she says what is happening on the ward. I need to sort it out and solve it myself. […] Yes, it has changed. Because… I really wasn’t easy. […] No. I was just full of anger. [...] as a flex worker you find yourself in different situations every time. [...] And one person can say never mind let it go, but the other you say today is a collision. I don’t want to be like that anymore. (20190924)

Through conversations with the work–life coach, she became more aware of how she was taking out her bad moods on the people in her work environment such as colleagues and patients, and that she wanted to stop behaving like this. Other examples of changes in social behaviors, specifically in working life, were to resume work after a period on sick leave, apply for a new job, and to set new priorities concerning work and overtime. Participants told us that the topic of menopause and midlife is now discussed between colleagues more often. Finally, in terms of changes in social behavior specifically related to private life, participants started to make changes in regard to planning, such as a less busy schedule. On the advice of the menopause consultant, a participant with young children called her family together to tell them what the menopause was doing to her, which would potentially help her to gain more understanding from within the family.

#### 3.2.2. Changes in Physical Health

Participants stated that participation in the WLP had reduced various physical symptoms. All these symptoms were associated with the menopause, and are included as menopausal symptoms in GCS under somatic and vasomotor symptoms. The participants reported that they had experienced a reduction in symptoms ranging from breathing difficulties to muscle and joint pains. To illustrate this, we use two quotes from a 54-year-old patient food services assistant. Her job involved physical work on a fulltime basis and she had been on sick leave for almost a year. She had experienced a number of physical health symptoms, including asthma. In the WLP, she learned how to perform breathing exercises through an application on her phone. In the following quote, she reflects on how these exercises promoted change:

Before I go to sleep I do the exercise for maybe ten minutes or fifteen minutes. At the start I did five minutes, or maybe not even four minutes, I was really tired. I couldn’t carry on. I really had to get out of bed and have a drink of water. But now, although it has taken some time, I have been doing it now … what…. almost six, seven, eight weeks. […] And thanks to doing her exercises, I don’t need to use my inhaler as much. I used to use it seven times a day and now it is only three. […] I am wondering that if I, continue if maybe I will get down to using it only once, or maybe not at all. My aim is to not use it at all. (20200123)

Besides this improvement in her ability to breathe, she also had less joint pain. In the following quote she reflects on what may have initiated this improvement:

Maybe it is because of those, those vitamin tablets. And also my food—I have started eating more vegetables. And more nuts. I used not to eat those much. So that kind of thing, that diet wheel that has, that helps too. And I think it has more benefits. Because I was really in pain. I really couldn’t... (20200123)

This participant started using dietary supplements in combination with a calcium-rich diet on the advice of the menopause consultant. These quotes suggest that the WLP supported the making of healthy choices and improved physical health.

#### 3.2.3. Changes in Mental Wellbeing

In almost all the interviews, participants reported changes in mental wellbeing concerning work and private life to a greater or lesser extent. Reported changes in mental wellbeing included feeling less stressed, less depressed, less tired, and having more energy due to sleeping better. These symptoms in which women felt changes are also associated with menopausal symptoms and are included in the GCS as psychological symptoms.

Participants also mentioned changes in mental wellbeing concerning work–life balance and work functioning. One participant said that she now enjoys going home from work more, and she relates this partly to the fact that she is more open to patients at work and deals differently with them. One participant also said that she now comes home from work less tired, because she has learned to protect her personal boundaries. She has now passed on various tasks to colleagues. With regard to an improvement in work functioning, participants reported improved interaction with colleagues, feeling fitter at work as result of a less busy private schedule, and being less tense.

#### 3.2.4. Changes in the Workplace

For some of the participants, the support in the WLP led to positive changes in the workplace, such as more openness about the topic of menopause and midlife, or a decrease in heavy workload. A 48-year-old patient food service assistant had a very heavy workload on the hospital ward where she works. The WLP supported her in starting a conversation with her manager about her heavy workload, as she realized she could hand over work to others:

She also taught me that I should try, also try, must try to hand over work to someone else. I’m, I always want to do everything myself, but she says, she said to me: you have to try to hand things over to someone else. Then you feel lighter in your head too, so I went to my manager and told her. And now she has assigned people, they are fellow students, they don’t actually do much on the ward. And they, they may help me, or they have to help me. It is in their job description. She has hung up a list with their job description, and they help me. And I walk up to them, and then I ask very politely if they can help with this or… you know what I mean? So, it is a success, yes it is. (20191127)

This conversation with her manager led to her being given more support in her work, and even resulted in the hiring of an extra colleague. Her tasks have been structurally eased as a result of this conversation.

## 4. Discussion

The results of our study indicate that the female workers in low-paid jobs experience a positive impact from the WLP. In terms of our five outcome variables, the quantitative analysis indicates a significant benefit in terms of menopausal symptoms only. However, the qualitative analysis of the perceived impact of the WLP on health and work functioning indicates that the WLP initiated a process of mental empowerment in female workers in low-paid jobs that has a positive impact on the participants’ behavior, physical health, mental wellbeing and the workplace.

The positive impact of the WLP on menopausal symptoms can be explained by the fact that women were encouraged to openly speak about their personal issues related to midlife. The different components of the WLP were focused on these issues—participants mentioned that they finally felt recognition for what they are going through and felt less worried. In addition, participants were provided with tools for influencing certain behaviors and situations. Participants have actually started making new choices that benefit their health and well-being. The results of the quantitative analysis in terms of participants experiencing a positive effect on menopausal symptoms after following the WLP are in line with two other studies on workplace health promotion interventions for female workers in menopause [29,30]. However, in these studies an integral approach was lacking—they focused mainly on menopause and work and not on private life or health behavior changes, for instance. Furthermore, these studies were mainly focused on different types of female workers, and to our knowledge, our study is the first to look at female workers in low-paid jobs. Reaching and engaging with this group in workplace health promotion interventions as well as in research is challenging [54]. The improvement in menopausal symptoms in this group appears promising, as according to the quantitative outcome variables, the WLP was partially effective. Interventions, such as the WLP, which take an integral approach to support menopausal female workers during midlife, have hardly been studied at all; a recent literature study acknowledged that it is worthwhile to study such interventions in other professions, sectors, and at different hierarchical levels (15).

With the exception of the significant improvement in menopausal symptoms seen in our quantitative results, and despite having enough power, we saw no effect in the outcome variables. We have two possible explanations for this. First, the WLP concerns a health promotion intervention, which allowed all women in the target group to participate, thus not solely women with severe symptoms or problems at work. At baseline, the mean score of NFR was 43.4. Workers with a high NFR had a total score of ≥55 (1–100 scale) and were at higher risk of chronic physiological stress reactions [55], prolonged fatigue [56], work-related fatigue [6], subjective ill health [57], and depression [58]. The baseline mean score was below that number. We calculated that 50.0% of the sample had a higher risk of psychological problems at baseline. However, the SD was 31.61 which means that the scores were spread out over a large range of values. We might have had more impact if we had pre-selected women who already had problems in work functioning or quality of life. If participants had been pre-selected on having problems, there would have been more room for improvement. This also applied to the improvement in menopausal symptoms, which had only a small effect (effect size 0.39), which makes us wonder if this is a clinically relevant effect. However, taking into account that the level of bothersome menopausal symptoms at baseline was not high—for instance, only 14.7% of the participants experienced very severe hot flashes and 15.7% very severe night sweats—the finding of a statistically significant effect does indicate relevant changes.

A second possible explanation for the lack of effect on outcome variables is that improving work functioning is difficult to achieve among female workers in low-paid jobs, especially within the short timeframe of our study. It could be that the WLP is merely intervening on the individual level of the employee, which might not be enough to improve problems with work functioning. What type of accommodation to an individual intervention such as the WLP would be required to resolve this issue? The next step in strengthening this type of intervention is to complement them with interventions at the organizational level. Various suggestions have already been made in the literature for interventions at the organizational level that support menopausal female workers [1,8,14,27,59]. We think that the next logical step is to start to increase awareness of issues related to midlife and menopause in the workplace among professionals such as occupational health physicians, human resources staff and line managers. With regard to improving problems in work functioning, it seems particularly relevant to educate line managers on this topic, and to improve their communication and behavioral skills [27]. This would enable line managers to recognize problems and start a conversation with their employees that could help in offering support. Hence, interventions at the organizational level could be examined to gain insight into the role of employers in improving problems in work functioning among female workers in low-paid jobs.

Despite the lack of effect, the results from the qualitative analysis indicate that the WLP empowers female workers in low-paid jobs to make choices that enhance their health and wellbeing at work and in their private lives, as summarized in the quote of one participant: “I get that spirit in me!” In making these new choices, the majority of participants feel more stronger or more free. However, this result is in contrast to our outcome in anxiety in the GCS, where we found no significant improvement. This may possibly be explained by the fact that the experiences with regard to feeling more free are different from those concerning the anxiety symptoms in the GCS. Although not for everyone, this process of mental empowerment initiated positive changes in four domains: behavior, physical health, mental wellbeing, and in the workplace. First, participants provided a wide range of examples of how they started to change their behavior with regard to lifestyle, health and in social relationships. Such behavioral changes include doing physical and breathing exercises more frequently in order to keep up with physical workload, learning to start a conversation with a partner about a personal issue such as loss of interest in sex and creating a less busy private schedule in order to have more time for oneself. Second, participants experienced positive changes in physical health. Third, participants experienced positive changes in mental wellbeing. Fourth, some participants have also experienced positive changes in workplace characteristics, such a decrease in workload. Therefore, according to our qualitative analysis, the WLP has brought about more than merely an improvement in menopausal symptoms, but has also started a process of mental empowerment which has made changes in different domains related to health and work functioning.

Since our participants were female workers in low-paid jobs with diverse migratory backgrounds, a mixed-method design in which we collected quantitative and qualitative data has been very helpful. The WLP is an intervention with an integrated approach aimed at supporting female workers in many different facets of their midlife. In the semi-structured, in-depth interviews, there was room for in-depth discussion on a wide range of subjects that are hard to examine statistically. Without these qualitative methods, including purposive sampling, we probably would not have been able to gain insight into what the WLP has done for a great variety of female workers in this target group. For example, we have learned that the WLP has encouraged this group of workers to engage with their line manager in order to reduce workload, how the topic of midlife and menopause is more openly discussed with the social environment, and how these workers feel fitter and less tense at work. These kinds of examples are not reflected in the pre-and post-test results. The interviews highlighted that the experiences of why and how participants made new choices, and what these choices resulted in are highly personal depending on factors such as cultural values, gendered behaviors and type of job. At group level, the WLP does not seem to have had an impact on the outcome variables, but the interviews have shown that female workers have made progress in health and wellbeing at work and in their private lives in their own particular ways.

When generalizing our results to other populations, a number of uncertainties should be considered. Firstly, we used self-reported validated questionnaires and included participants with a migratory background who were not proficient in Dutch. The support of an interpreter may still not have been sufficient. Secondly, the questionnaires were not cross-culturally adapted. Cross-cultural adaption is based on the idea that measures used across cultural groups should not only be translated, but also be culturally accommodated to maintain content validity of the instrument at the conceptual level [60]. Thirdly, there was a large cultural diversity in our study population in a relatively small sample. At pre-test (t0), more than half of the participants in this study had a migratory background, with 21 different nationalities. Although variation in the qualitative study helped to enrich the data, in quantitative studies with a relatively small sample size this leads to a wide spread in confidence intervals. Fourthly, the sample size of both the quantitative and qualitative study was relatively small and precludes drawing firm conclusions. Fifthly, when interpreting the results of our qualitative analysis, we also acknowledge a degree of selection bias in terms of the participants with whom we conducted the semi-structured, in-depth interviews. These participants took part voluntarily and were possibly more positive towards the WLP than other potential participants may have been. Since all participants received an economic incentive, a degree of selection bias does also apply to participation in both the quantitative and qualitative study. Sixthly, although less suitable for a non-standardized intervention in a heterogeneous study population, the lack of a control group precludes drawing firm conclusions of the quantitative analysis. For these reasons, the results must be interpreted with caution and may only be generalized to the wider population of women in low-paid jobs.

## 5. Conclusions

This study is the first scientific evaluation of a workplace health promotion intervention aimed at integrally supporting female workers in low-paid jobs during menopause and midlife. While our data does not justify firm conclusions, our findings suggest that female workers in low-paid jobs experience a positive impact from the WLP. The WLP is an intervention that empowers female workers in low-paid jobs to make choices that benefit their health and wellbeing, both at work and in their private lives, as summarized in the quote of one participant: “I get that spirit in me!” In addition, we have come to the conclusion that additional qualitative methods are indispensable in evaluating the impact of an intervention aimed at integral support among a very heterogeneous study population.

However, it is not yet clear how problems in work functioning and quality of life can be improved at group level. Future research should focus on an evaluation of the WLP in a larger study sample, in particular if women across different migratory backgrounds are to be included, with a longer follow-up. Furthermore, future research should also focus on interventions at the organizational level, how employers can play a role in improving problems in work functioning and the quality of life among female workers in low-paid jobs during menopause and midlife. At last, we now know that participating in the WLP raises the spirits of these workers; in future studies, we could examine if the WLP or a similar type of intervention is able to reduce sickness absence.

## Figures and Tables

**Table 1:** Topic list semi-structured, in-depth interviews.

**Lifestyle**
*Perceived changes*
Tell me whether you experienced any changes in your body, exercise habits, or nutrition after following the work-life program (WLP).
Tell me what has changed. Tell me how this has changed. Tell me why nothing has changed.
*Contribution WLP*
Tell me how and if the WLP contributed to these changes.
**Health**
*Perceived changes*
Tell me whether you experienced any changes in your health – menopausal symptoms, physical- and mental health—after following the WLP.
Tell me what has changed. Tell me how this has changed. Tell me why nothing has changed.
*Contribution WLP*
Tell me how and if the WLP contributed to these changes.
**Work functioning**
*Perceived changes*
Tell me whether you experienced any changes in your work after following the WLP.
Tell me whether you experienced any changes in your work-life balance after following the WLP.
Tell me what has changed. Tell me how this has changed. Tell me why nothing has changed.
*Contribution WLP*
Tell me how and if the WLP contributed to these changes.

**Table 2:** Participant characteristics (*n* = 70, including drop-outs).

Descriptive	*n*	(%)	Mean	SD
Age			52.6	4.5
Ethnicity				
Ethnic minority	36	(51.4%)		
Ethnic majority (Dutch)	34	(48.6%)		
Educational level				
Low	29	(41.4%)		
Intermediate	36	(51.4%)		
High	5	(7.1%)		
Subjective social status (SSS) ^1^			4.7	1.5
Living situation				
Alone	11	(15.7%)		
With partner	11	(15.7%)		
With partner and children	32	(45.7%)		
No partner but with children	15	(21.4%)		
Other	1	(1.4%)		
Main wage earner				
Yes	38	(54.3%)		
No, my partner	24	(34.3%)		
Equal with partner	8	(11.4%)		
Type of contract				
Full time	22	(31.4%)		
Part time	48	(68.6%)		
Informal care responsibilities				
Yes	15	(21.4%)		
No	55	(78.6%)		
Menopausal status ^2^				
Pre-menopause	4	(7.1%)		
Early peri-menopause	3	(5.4%)		
Late peri-menopause	3	(5.4%)		
Post-menopause	38	(67.9%)		
Unknown	8	(14.3%)		

^1^ 1 missing. ^2^ Menopause status is determined through t1 (*n* = 56).

**Table 3:** Outcomes on pre-test–post-test, paired *t*-tests and Wilcoxon signed-rank test results, and effect sizes.

	*n* ^2^	T0 (Pre-Test) ^3^		*n* ^2^	T1 (Post-Test)		Pre- and Post-Test			Effect Size
		Mean (SD)	Median (IQR)		Mean (SD)	Median (IQR)	Mean Diff ^4^	*t* or *z* ^5^	*p*	
Work functioning problems (0–100) (lower is better)	65		21.1 (20.0)	54		18.6 (16.0)		*z* = −0.27	0.791	
Work performance	70		00.0 (6.0)	55		00.0 (6.0)		*z* = 0.59	0.556	
Need for recovery after work ^1^	70		40.9 (55.0)	55		36.4 (64.0)		*z* = −0.66	0.508	
Quantity of work	66	21.4 (19.88)		54	20.8 (16.68)		2.4	*t* = 0.94	0.350	
Capacity to work	69		18.8 (27.0)	55		19.6 (25.0)		*z* = 0.99	0.320	
Menopausal symptoms (GCS) (0–63) (lower is better)	70		18.0 (12.0)	56		13.0 (11.0)		*z* = −3.91	0.000	0.39 (0.03, 0.74)
Psychological	70		8.5 (8.0)	56		7.0 (7.0)		*z* = −2.59	0.010	0.30 (−0.05, 0.66)
Anxiety	70	4.8 (3.10)		56	4.4 (2.73)		0.5	*t* = 1.68	0.099	
Depression	70		4.0 (4.0)	56		3.0 (4.0)		*z* = −3.08	0.002	0.41 (0.05, 0.76)
Somatic	70	5.4 (3.04)		56	4.3 (2.81)		1.1	*t* = 3.47	0.001	0.35 (−0.00, 0.71)
Vasomotor	70		2.0 (3.0)	56		2.0 (3.0)		*z* = −3.24	0.001	0.33 (−0.02, 0.68)
Sexual dysfunction	70		1.0 (1.0)	56		0.0 (1.0)		*z* = −0.78	0.438	
Quality of life (0–100) (higher is better)										
Physically	70		48.8 (15.0)	56		50.1 (10.0)		*z* = −1.15	0.250	
Mentally	70	48.2 (10.37)		56	48.7 (9.40)		−0.2	*t* = −0.12	0.908	
Work ability (0–10) (higher is better)	70		7.0 (1.0)	56		8.0 (1.0)		*z* = −1.80	0.072	

^1^ Primary outcome variable. ^2^ The *n* varies due to missing values on the outcomes. ^3^ The outcome measures vary depending on which test is used. ^4^ Mean differences based on complete data sample (t0 + t1). ^5^ Either *t* or *z* vary depending on which test is used.

**Table 4:** Characteristics of interview participants (*n* = 12).

Descriptive	*n*	(%)	Mean	SD
Age			53.4	4.2
Ethnicity				
Ethnic minority ^1^	7	(58.3%)		
Ethnic majority (Dutch)	5	(41.7%)		
Educational level				
Low	4	(33.3%)		
Intermediate	8	(66.7%)		
High	0	(0.0%)		
Type of contract				
Full time	6	(50.0%)		
Part time	6	(50.0%)		
Living situation				
Alone	2	(16.7%)		
With partner	3	(25.0%)		
With partner and children	6	(50.0%)		
No partner but with children	1	(8.3%)		
Menopausal status				
Pre-menopause	1	(8.3%)		
Early peri-menopause	1	(8.3%)		
Late peri-menopause	0	(0.0%)		
Post-menopause	9	(75.0%)		
Unknown	1	(8.3%)		

^1^ Ethnic backgrounds included Brazil, Guinee, Morocco, Portugal, and Surinam. The quote “I get that spirit in me” is from a participant from Ghana with whom the first author held a pilot group interview before the twelve interviews included in this article.
